# Deep learning-assisted survival prognosis in renal cancer: A CT scan-based personalized approach

**DOI:** 10.1016/j.heliyon.2024.e24374

**Published:** 2024-01-12

**Authors:** Maryamalsadat Mahootiha, Hemin Ali Qadir, Davit Aghayan, Åsmund Avdem Fretland, Bjørn von Gohren Edwin, Ilangko Balasingham

**Affiliations:** a The Intervention Centre, Oslo University Hospital, Oslo, 0372, Norway; b Faculty of Medicine, University of Oslo, Oslo, 0372, Norway; c Department of Electronic Systems, Norwegian University of Science and Technology, Trondheim, Norway

**Keywords:** Cancer prognosis, Renal cell carcinoma, Kidney tumor grading, Survival analysis, Deep learning, Personalized prognosis, Imaging biomarkers, Radiomics

## Abstract

This paper presents a deep learning (DL) approach for predicting survival probabilities of renal cancer patients based solely on preoperative CT imaging. The proposed approach consists of two networks: a classifier- and a survival- network. The classifier attempts to extract features from 3D CT scans to predict the ISUP grade of Renal cell carcinoma (RCC) tumors, as defined by the International Society of Urological Pathology (ISUP). Our classifier is a 3D convolutional neural network to avoid losing crucial information on the interconnection of slides in 3D images. We employ multiple procedures, including image augmentation, preprocessing, and concatenation, to improve the performance of the classifier. Given the strong correlation between ISUP grading and renal cancer prognosis in the clinical context, we use the ISUP grading features extracted by the classifier as the input to the survival network. By leveraging this clinical association and the classifier network, we are able to model our survival analysis using a simple DL-based network. We adopt a discrete LogisticHazard-based loss to extract intrinsic survival characteristics of RCC tumors from CT images. This allows us to build a completely parametric survival model that varies with patients' tumor characteristics and predicts non-proportional survival probability curves for different patients. Our results demonstrated that the proposed method could predict the future course of renal cancer with reasonable accuracy from the CT scans. The proposed method obtained an average concordance index of 0.72, an integrated Brier score of 0.15, and an area under the curve value of 0.71 on the test cohorts.

## 1 Introduction

Renal cancer is one of the most frequently diagnosed forms of cancer affecting the kidneys, with a significant yearly increase in its incidence and mortality rates. In 2020, it was estimated that there were a total of 431,288 new cases of renal cancer and 179,368 related deaths affecting individuals of both genders [1]. Cancer, in general, is a hazardous disease, and the treatment requires a comprehensive and multidisciplinary approach. Therefore, it is time-consuming, complex, and costly, requiring extensive medical procedures. For instance, in the case of renal cancer, surgical removal is still the most widely used treatment option for localized tumors [2]. Recently, new targeted therapies for treating renal cancer have been introduced to enhance patient outcomes and reduce the need for surgical interventions [3], [4]. However, patients can experience different survival times and respond differently to various treatment options based on their covariates and the tumor's biological behavior and characteristics.

Renal cell carcinoma (RCC) is the most common type of renal cancer. The assessment of the RCC's grade is a crucial factor in predicting a patient's survival and response to treatment [5], [6]. Until recently, Fuhrman grading [7] was RCC's most widely used grading system. However, doubts have been raised about the usability of the Fuhrman grading system for renal cancer prognosis [8]. To address this issue, the International Society of Urological Pathology (ISUP) introduced a new RCC grading system, the ISUP grading classification, in 2012. The ISUP grading categorizes RCC into four grades: 1, 2, 3, and 4, based on the microscopic morphological changes in histological patterns [6].

The ISUP grading of RCC is determined by examining pathological parameters obtained from biopsy and resected specimens, which requires experienced pathologists and is an invasive process. However, recent studies have demonstrated the possibility of obtaining microscopic morphological patterns from the appearance of tumors through preoperative analysis of cross-sectional imaging modalities such as computed tomography (CT) scans or magnetic resonance imaging (MRI) [9], [10]. Despite the advantages of this approach, manual interpretation and assessment of radiological data can be challenging and imprecise, prone to human error and limitations in perceiving hidden information.

In recent years, machine learning in the form of Deep Learning (DL) has significantly contributed to the automation of renal cancer classification and staging [11], [12], [13], [14], [15], [16], [17], [18], [19]. DL has demonstrated its ability to find complex hidden patterns within a set of training data and convert raw input images into meaningful features. Most DL-based RCC-subtype classification models have been developed to examine whole-slide histological images of kidney tumors [11], [12], [13]. These models aimed to differentiate the microscopic morphological patterns into various subtypes of RCC, such as Clear Cell RCC, Papillary RCC, Chromophobe RCC, and Renal Oncocytoma. Contrarily, the potential of DL in processing radiological data has been explored by various studies for automatic RCC-subtype classification [14], [15], [16], [17] and staging [18], [19]. A substantial number of DL models have been proposed for the classification of renal tumors as benign or malignant using either CT [17], [15] or MRI [14], [16] images. Recently, our group conducted a study investigating the feasibility of utilizing DL for RCC grade classification from CT scans [20]. The outcomes from this study demonstrate that DL can comprehend tumor patterns within radiological data and correlate them with the RCC categories, stages, and grading.

Unlike the previous studies, in this paper, we investigate the feasibility of DL in modeling survival analysis for patients diagnosed with renal cancer. Our hypothesis is that DL can extract prognostic patterns from the surface of RCC tumors depicted in 3D CT data. To verify this hypothesis, we present a DL-based approach that computationally predicts individual survival probability curves for each patient from their CT scans that can be available before undergoing invasive procedures such as surgery. Our proposed method consists of two sub-networks: a classifier- and a survival- network. The classifier network takes in a 3D CT scan and processes it to extract features relevant to ISUP grading classes, ultimately predicting the final ISUP grade of the RCC tumor present in the scan. Given the strong correlation between ISUP grading and prognosis, as stated in the previous study [6], we use the same extracted features from the classifier network as input to the survival network, which then predicts the survival probability over time. Our experimental results show that our DL-based model can effectively predict the future course of renal cancer with reasonable accuracy solely based on the CT scans. However, the model's performance in a real-time clinical setting requires prospective validation with a larger and more diverse patient cohort.

## 2 Related works

The Cox proportional hazards (CPH) model, a classical survival model approach that considers censored times, has been extensively utilized in research [21]. CPH is a linear model assuming a proportional relationship between the input variables and survival times and cannot capture non-linear relationships. Researchers have explored the use of DL models to address the linearity issue in survival analysis.

Ching et al. [22] and Katzman et al. [23] introduced Cox-nnet and DeepSurv, respectively. Cox-nnet and DeepSurv used neural networks and employed Cox regression as the loss function. Both models were tested with gene expression and clinical data. Cox-nnet and DeepSurv models have been widely used as a foundation for various other studies that aim to address the linearity issue of the CPH model; in the following, we review several of these studies.

Mobadersany et al. [24] predicted cancer prognosis using histological images and genetic biomarkers. Their model employed Convolutional neural network (CNN) layers and fully connected (FC) layers with Cox loss to estimate survival times. Wang et al. [25] created a CT-based DL model for recurrence analysis. They used a convolutional autoencoder for extracting the CT image features and supplied it with cropped CT tumor regions. They utilized the Cox hazard regression to predict prognosis with image features as the inputs. Courtiol et al. [26] predicted survival with histopathological images. They used deep CNNs with the Cox regression. Hao et al. [27] developed PAGE-Net to predict cancer prognosis using genetic data and histopathological images. They integrated CNNs for histopathology images and FC layers for genetic data using the Cox loss function in the output layer. Mukherjee et al. [28] created LungNet to predict patient survival periods from lung CT data with a shallow CNN using Cox and cross-entropy losses. Wu et al. [29] and Zhang et al. [30] have contributed to cancer prognosis research using clinical data and CT images. Wu et al. applied a 3D ResNet for feature extraction and use MSE for survival prediction in non-small cell lung cancer, while Zhang et al. utilized 2D convolutional layers for feature extraction and used customized loss for gastric cancer prognosis. Zhong et al. [31] used MRI and clinical variables to predict the cancer prognosis. They used CNNs to analyze images and Cox regression to train the survival model. Chen et al. [32] suggested predicting cancer prognosis using radiological imaging, gene expression, and clinical risk factors. They employed autoencoders as a means to regenerate genomic features for gene expression analysis. In their proposed architecture, a single layer integrated gene features, radiological imaging features, and clinical data to produce survival curves based on the Cox regression.

Although models based on Cox-nnet and DeepSurv attempted to solve the linearity problem in survival analysis, they could not address the issue of proportionality. Recent approaches employed a completely parametric approach to solving proportionality issues that involved discretizing times into specified time intervals and outputting predictions for these intervals. Fotso et al. [33] employed logistic regression while the authors of Deephit [34] parameterized the probability mass function of the survival distribution and added a ranking component to the loss. Another strategy for solving proportionality involved calculating each period's discrete conditional hazard rate. This concept was first established decades ago [35] and was recently applied to contemporary DL approaches known as Nnet-survival [36]. Recently, Vale-Silva and Rohr [37] introduced MultiSurv as a non-linear and non-proportional cancer prognosis. MultiSurv utilized clinical data, omics data, and histopathology images and created a fusion layer from feature vectors that came from different modalities.

## 3 Our contributions

This study aims to enhance patient survival probability prediction from CT scans by employing a non-linear and non-proportional method. We present the initial implementation of a personalized survival estimation framework for renal cell carcinoma patients, utilizing a DL methodology applied to computed CT scans. Our survival model possesses a number of advantages over the prior works: 1) we incorporate 3D inputs and 3D convolutional layers to avoid losing any information related to the boundary between the tumor and healthy tissue. 2) We use a discrete LogisticHazard-based loss in our survival model instead of CPH, which enables us to predict non-proportional survival probability curves for different patients, providing a more accurate reflection of reality than previous studies. 3) Despite relying solely on CT scans, our survival model is able to attain a high C-index. 4) The output of our survival model is personalized for each patient, producing a unique survival probability curve per patient. 5) Similar to the clinical studies that have associated ISUP grades with mortality and survival rates, we use ISUP grading for prognostic feature extraction from CT volumes for our survival analysis and evaluate this association computationally.

## 4 Methodology

**Figure 1:**
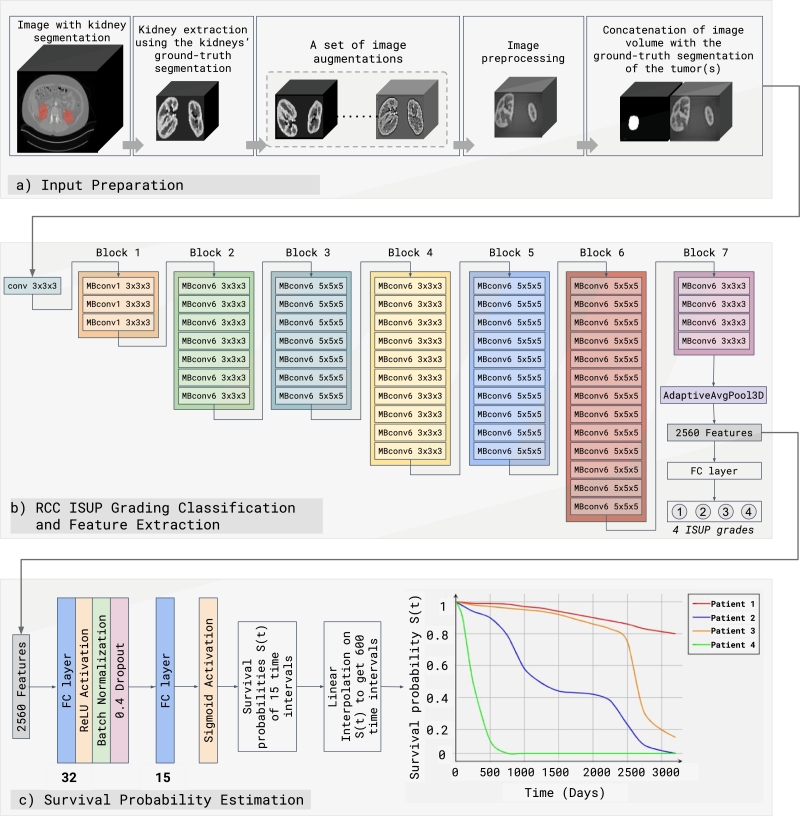
Our proposed methodology has three key stages. Stages *a* and *b* target kidney tumor classification, while stage *c* estimates survival. Stage *a* primarily involves image preparation for network training using various techniques. We extract the kidneys from full abdomen images using the provided kidney segmentations. We employ multiple image augmentation methods to overcome the imbalance issue in the dataset and the limited number of samples for training. We apply some image preprocessing techniques to enhance the quality of the image. The final step in stage *a* is merging the tumor with the image on the channel dimension to compel the classifier model to focus on the size and position of the tumor. We adapt Efficient-Net B7 to classify the tumors in stage *b*. The primary block of Efficient-Net B7 is MBconv, a convolutional layer. MBConv1 and MBConv6 are various MBConv versions. The key distinction between MBConv1 and MBConv6 is the size of the convolutional kernels and the number of layers. After MBConvs, one Adaptive Average Pooling layer and several fully connected layers are utilized to classify images into four ISUP grades. From the Adaptive Average pooling layer, CT image features are extracted. The extracted features are utilized as input to a neural network comprising two fully connected layers. The first layer contains 32 neurons and employs the ReLU activation function, followed by batch normalization and 0.4 dropouts. The second fully connected layer consists of 15 neurons and utilizes a sigmoid activation function to produce the final output. We train the survival model with LogisticHazard-based loss. The survival probabilities for 15 time points are estimated for each patient. Linear interpolation is used to generate survival probabilities of 600 time points, and finally, we can depict the patient's survival curve.

Fig. 1 exhibits our novel DL-based approach to estimating each patient's personalized survival probability curve. Our methodology is divided into three main stages. The first stage involves the preparation of input data, as illustrated in part (a) of Fig. 1. Subsequently, the methodology encompasses two DL-based networks, which are detailed in parts (b) and (c) of Fig. 1. Specifically, the network depicted in part (b) of Fig. 1 is designed for the classification of RCC tumors into ISUP grades. Meanwhile, the network shown in part (c) of Fig. 1 is dedicated to survival analysis. To ensure accuracy, we train these two networks separately. The ISUP grading network serves as the prognostic feature extractor for the survival network, where the extracted features are utilized as inputs to predict personalized survival probability curves.

### 4.1 Input preparation

Before proceeding with training our two DL-based networks, it is beneficial to pre-process the input CT volumes to prepare the images for optimal performance during training. The pre-processing stage involves several steps, including kidney extraction, data augmentation, data preprocessing, and tumor concatenation. Fig. 1, part *a*, shows the input preparation phase in detail.

We extract the left and right kidneys from the whole abdomen CT volumes using the provided annotations of kidney segmentations. The dataset we use for this study has an imbalance issue, as outlined in 5.1, due to the varying number of patients in each ISUP class. In addition, 244 samples might not be sufficient to train a deep neural network from scratch for an image classification model. Studies have suggested that training a smaller network from scratch could be more beneficial than transferring knowledge from natural images to medical images [38]. We apply several data augmentation strategies to overcome the class imbalance issue and the small number of samples for training our ISUP grading classifier from scratch. Leveraging the capabilities of the MONAI library, we have employed MONAI transformers^1^ for our data augmentation process.

Our dataset originally contained an imbalanced distribution of ISUP grades, with 33 ISUP1 patients, 119 ISUP2 patients, 66 ISUP3 patients, and 26 ISUP4 patients. We utilized class-aware data augmentation, where the augmentation factor differed per ISUP grade. Augmentation was tailored to produce around 500 samples for the under-represented ISUP1, ISUP3, and ISUP4 classes while limiting the augmentation of the majority ISUP2 class. This variable augmentation approach led to balanced class distributions within each split, though with different total samples due to the customized per-class factors. Table 1 shows the resulting sample sizes for the training, validation, and test sets after augmentation. Our class-aware augmentation strategy enabled more robust model training and evaluation compared to using the imbalanced original dataset.

**Table 1:** Number of training, validation, and test set after augmentation.

Fold	Training	Validation	Test
**1**	1150	184	659
**2**	1133	184	676
**3**	1151	184	658

Within our augmentation strategy, we have specifically utilized position and noise transformers, including affine, rotate, flip, Gaussian noise, space spike noise, and Gibbs noise. As illustrated in Table 2, various augmentation techniques are employed in this study. Affine transformations, particularly shear operations, are set to parameters (0, 0.5, 0), mainly inducing shear along the y-axis, creating variations in orientation horizontally. Both horizontal and vertical flipping are conducted with a 50% probability. Random rotations are introduced within a range of -90 to 90 degrees. Gaussian noise is integrated with a mean of 0 and a standard deviation of 1. Additionally, SpaceSpike noise is added, with intensity values ranging from 10 to 13. The selection of this range is based on preliminary hyperparameter tests, where intensities from 5 to 15 are assessed. The 10-13 range is deemed optimal as it ensures varied augmentation without causing significant distortion. Lastly, Gibbs noise is consistently applied to all augmented images with a probability of 1. The alpha values for Gibbs noise are set between 0.6 to 0.8. This range is selected based on empirical observations, ensuring the presence of noticeable speckle noise but also preserving the quality of the image. The noise augmentations are additional noises applied for augmentation only, and the purpose is to increase diversity and improve generalization.

**Table 2:** Data augmentation techniques and implementation details.

Augmentation Technique	Values Used
Affine Shear	(0, 0.5, 0)
Horizontal Flip	Probability 0.5
Vertical Flip	Probability 0.5
Rotation	(-90, 90) degrees
Gaussian Noise	Mean 0, Std Dev 1
SpaceSpike Noise	Intensity (10, 13)
Gibbs Noise	Alpha (0.6, 0.8), Probability 1

To improve the classification performance, we subject the images to several other preprocessing steps, which aim to both standardize and enhance the quality of the images. We resize the volumes to [128, 128, 128], reorient them to RAS,^2^ and normalize them based on the mean and standard deviation during the preprocessing steps. In the final step, the extracted and pre-processed kidneys are concatenated with the corresponding ground truth of the tumor segmentation annotations into a single tensor on the channel dimension. The aim of this concatenation is to prompt the ISUP grading classifier network to pay particular attention to the tumors' location, size, and surface pattern. The output of this stage served as the input of our RCC ISUP grading classification stage, discussed in detail in the following section.

For a deeper dive into the technical aspects of input preparation, such as image augmentation, preprocessing, and concatenation, we refer you to a separate study published as a conference paper [20]. This study covers the classification aspect in more detail.

### 4.2 RCC ISUP grading classification

In this stage, we classify the RCC tumors presented in the pre-processed input into ISUP grades. Given a strong relationship between the ISUP grading and its applications in detecting recurrence, progression, and mortality in the literature [5], [6], retrieving features from the ISUP grading classifier network can be significant for RCC survival estimation. As a result, these learned features can be used as the input of our second network, which is responsible for predicting survival probability, as discussed in detail in 4.4.

#### 4.2.1 The classifier network

Fig. 1 part (*b*) shows the process of ISUP grading classification. We adopt the Efficient-Net B7 architecture as our classifier network since it is one of the most powerful CNN-based architectures available for image classification [39]. We selected EfficientNet-B7 as the backbone for our model due to its state-of-the-art accuracy results on computer vision datasets like ImageNet, outperforming popular models such as ResNet [40], Inception [41], and NasNet [42] while using fewer parameters and FLOPS [39]. Efficient-Net B7 employs Mobile Inverted Bottleneck Convolution (MBConv) blocks. MBConv is aimed to reduce computation and memory costs while retaining excellent classification accuracy [43]. We modify the structure of Efficient-Net B7 and convert it to a 3D CNN model so that it can process 3D image data such as CT volumes. As an output, the model generates probabilities for the four possible ISUP grades (1, 2, 3, and 4).

#### 4.2.2 Training the classifier network

As discussed in 5.2, we use three-fold cross-validation to train the classifier network. In each fold of the training process, we calculate the mean area under the receiver operating characteristic curve (AUC) to find the best network parameters. Mean AUC is computed after every training epoch on the validation set. When a new best mean AUC is found, the model parameters are saved, overwriting the previous model. We train the classifier network for 50 epochs, as further training iterations do not significantly reduce training loss. To quantify the loss, we use the Cross-Entropy loss, defined as Eq. (1):

(1) $$L=-\sum_{i=1}^{n}t_{i}\times log(p_{i}),$$

where $t_{i}$ is the true label, $p_{i}$ is the predicted softmax probability for the *ith* class, and *n* is the number of classes, which is 4 in this study. We use the ADAM optimizer [44] and a learning rate of $1\times 10^{-4}$.

#### 4.2.3 Classifier model evaluation

The three-fold cross-validation leaves us with three trained classifier networks. We assess the performance of our trained classifier networks based on accuracy, recall, and F1-score from Eq. (2), Eq. (3) and Eq. (4) respectively:

(2) $$\text{Precision}=\frac{\text{TP}}{\text{TP}+\text{FP}},$$

(3) $$\text{Recall}=\frac{\text{TP}}{\text{TP}+\text{FN}},$$

(4) $$\text{F-score}=2\times \frac{\text{Precision}\times \text{Recall}}{\text{Precision}+\text{Recall}},$$

where TP is the true positive, FP is the false positive, and FN is the false negative. We calculate the F1-scores for each fold by first averaging the F1-scores of the four ISUP classes within the fold. Then, we take the average of these values for the three folds.

In our research, we chose to augment the test set for two primary reasons: 1) Medical images inherently display variability due to factors like patient positioning, scan quality, and tumor characteristics. Our augmentations were tailored to simulate these real-world variations rather than arbitrary distortions. By augmenting the test set, we aimed to more rigorously evaluate the model's robustness to these typical discrepancies found in scans. 2) Given the limited size of our dataset, augmenting the testing set can enhance the statistical stability of our results, mitigating the risk of results being skewed by a few challenging or outlier cases.

Table 3 shows the average precision, recall, and F1-score for all classes over three folds for the test data set. The best fold was second, with an overall average F1-score of 0.84. They are calculated from the confusion matrix, demonstrated in Table 4 for samples in the test set.

**Table 3:** Classification evaluation metrics for three folds.

Fold	1	2	3
Precision	0.61	0.84	0.68
Recall	0.62	0.85	0.66
F1-score	0.61	0.84	0.67

**Table 4:**
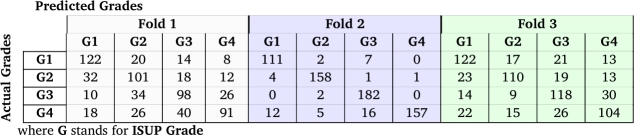
Confusion matrix of ISUP grading classification for different folds.

The variability seen in classification metrics among different folds is influenced by both the unique data distribution in each fold and our specific data augmentation approach. This variability suggests that our model might be more sensitive to particular data distributions. One factor that could explain the metric differences across folds is how the model responds to augmented images: if an original image is correctly classified, its augmented versions are likely classified correctly, too, and the same goes for misclassifications. The metrics across folds may be affected by the balance between original and augmented images. With a larger dataset or a varied distribution, we might see more consistent metrics across the folds. For a detailed discussion on the classification of images into ISUP grades, please refer to the paper by Mahootiha et al. [20].

### 4.3 Prognostic feature extraction

The classifier network receives 3D volumes as its input, and its task is to predict the ISUP grade class of each presented RCC tumor. Our classifier network consists of seven blocks of CNN layers, an Adaptive Average Pooling layer, and an FC layer. The adaptive average pooling layer serves as a bridge between the CNN layers and the FC layer, reducing the size of feature maps extracted from the convolutional layers to a compact, fixed-size representation, which can be fed into the FC layer for ISUP grading classification [40]. The adaptive average pooling layer enables spatially invariant feature extraction and mitigates overfitting by reducing model parameters [45]. Hence, the Adaptive Average Pooling layer is a promising choice for prognostic feature extraction, which can be employed as the input for the survival network.

To construct our prognostic feature vector for each patient, we retrieve the output of the average pooling layer after being flattened. We apply the trained classifier network on all patients in the used dataset to collect the prognostic features for every patient and subsequently store these prognostic feature vectors in a CSV file with 2560 columns and *M* rows, where *M* corresponds to the number of patients in the dataset. Before training the survival network, we normalize these features by mean and standard deviation.

### 4.4 Survival probability estimation

#### 4.4.1 Discrete time survival model

We aim to develop a fully parametric, non-linear, and non-proportional survival model. Neural networks can capture the non-linear relationship between variables and survival probabilities. However, when they are used with CPH, the proportionality of survival probability curves among the patients can not be solved. So, we tried to use LogisticHazard-based loss to solve the Cox hazard-based problems. The LogisticHazard model does not assume constant hazard ratios over time, unlike the Cox model which makes this assumption. This allows for more flexible modeling of non-proportional hazards. LogisticHazard regression can easily incorporate time-dependent covariates without needing complex modifications like stratification or time-splitting. This simplifies modeling time-varying effects. The LogisticHazard loss function handles tied events more robustly than the Cox partial likelihood when multiple subjects experience an event at the same time. This enhances reliability in the presence of tied event times.

We discretize the output of a neural network to predict survival probabilities for *n* time intervals. Creating a discrete-time survival model requires discretizing the continuous time interval by selecting an appropriate interval size. The interval size is obtained by dividing the maximum duration in the dataset by the number of intervals we want to have. So the patient's follow-up period is separated into *n* equidistant left-closed and right-open intervals. It is assumed that the influence of variables varies with time, resulting in time-varying hazard and survival probabilities. With this flexibility, it is possible to overcome the proportionality issue that plagued earlier models.

In the discrete-time survival model, conditional hazard probability varies from person to person. For a person that had an event during interval *j* (i.e., uncensored), according to Eq. (5), the likelihood is the probability of surviving through intervals 1 to j−1, multiplied by the probability of failing during interval *j*:

(5) $$likelihood=h_{j}\prod_{i=1}^{j-1}\left(1-h_{i}\right)\;,$$

which $h_{j}$ is the conditional hazard probability at the time of the event of interest, and $h_{i}$ is the conditional hazard probability before the time of the event. According to Eq. (6), the log-likelihood is:

(6) $$loglikelihood=\ln\left(h_{j}\right)+\sum_{i=1}^{j-1}\ln\left(1-h_{i}\right)\;.$$

For a censored person with a censoring time $t_{c}$ that occurs in the second half of interval j−1 or the first half of interval *j* (i.e., $\frac{1}{2}\left(t_{j-2}+t_{j-1}\right)\leq t_{c}<\frac{1}{2}\left(t_{j-1}+t_{j}\right))$, according to Eq. (7) the likelihood is the probability of surviving through intervals 1 to j−1:

(7) $$likelihood=\prod_{i=1}^{j-1}\left(1-h_{i}\right)\;,$$

which $h_{i}$ is the conditional hazard probability before the time of censoring. According to Eq. (8), the log-likelihood is:

(8) $$loglikelihood=\sum_{i=1}^{j-1}\ln\left(1-h_{i}\right)\;.$$

For every patient, based on the event of interest (dead or censored), the log-likelihood is calculated. The full log-likelihood of the observed data is the sum of the log-likelihoods of all patients in the dataset. The full log-likelihood is then utilized as the loss function for training a discrete-time survival model.

#### 4.4.2 Survival network

Fig. 1 part *c* presents our survival network architecture. The input to the survival network is the extracted feature vector from the classifier network. During the training of our survival network, the input size is a (p,f)-dimensional vector, where *p* is the number of patients, and *f* is the number of extracted features per patient, which is 2560.

Our proposed survival network comprises a simple architecture with two FC layers containing 32 and 15 neurons. The first FC layer is succeeded by a ReLU activation function, a batch normalization layer, and a dropout layer with a rate of 0.4. The second FC layer employs a sigmoid activation function to calculate survival probabilities for a range of time intervals.

Our survival network generates a (p,n)-dimensional vector, where *p* is the patch size (the number of patients), and *n* is the number of time intervals, for which we choose 15-time points in this study. The output vector comprises values corresponding to the estimated likelihood of survival within each specified time interval. Specifically, each component of the vector represents the predicted conditional probability of survival during a given time interval.

We utilize an interpolation technique proposed by Kvåmme et al. [46] to convert the predicted discrete-time survival estimates into continuous-time survival estimates. Specifically, we select 40 data points and perform a linear interpolation based on a constant density function within each interval.

#### 4.4.3 Survival network training details

We set the number of epochs to 500 and let the model use early stopping with a patience of 10. We choose Adam optimizer [44] and a learning rate of 0.001. We find an optimal learning rate using the method proposed by Smith [47]. The loss function according to Eq. (9) is a restatement of Eq. (6) and Eq. (8) after being vectorized for parallel processing:

(9) $$L=-\sum_{x=1}^{p}\sum_{i=1}^{n}\left(\begin{array}{c} \ln\left(1+{\mathrm{surv}}_{s}(x)(i)\cdot \left({\mathrm{surv}}_{\text{pred }}(x)(i)-1\right)\right)\\ +\ln\left(1-{\mathrm{surv}}_{f}(x)(i)\cdot {\mathrm{surv}}_{\text{pred }}(x)(i)\right) \end{array}\right),$$

where *p* is the number of patients, and *n* is the number of intervals. ${\mathrm{surv}}_{\text{pred}}(x)(i)$ is the output predicted probability of our survival model for patient *x* in the time interval *i* that can be a value between 0 and 1. Based on the ground truth, each patient has an established death event or censoring time *t*. For every patient, vector ${\mathrm{surv}}_{s}$ and vector ${\mathrm{surv}}_{f}$ are the ground truth events (can be 0 or 1), and both of them have length *n*. According to Eq. (10), Eq. (11) and Eq. (12), vector ${\mathrm{surv}}_{s}$ indicates the time intervals during which the patient has survived; vector ${\mathrm{surv}}_{f}$ indicates only the time interval during which death occurred. For patients with uncensored events, in the time interval *i*:

(10) $${\mathrm{surv}}_{s}(x)(i)=\left\{ \begin{array}{cc} 1, & \text{ if }t_{x}\geq t_{i}\\ 0, & \text{ otherwise } \end{array}\right.\;{\mathrm{surv}}_{f}(x)(i)=\left\{ \begin{array}{cc} 1, & \text{ if }t_{i-1}\leq t_{x}<t_{i}\\ 0, & \text{ otherwise } \end{array}\right.$$

for patients with censored events:

(11) $${\mathrm{surv}}_{s}(x)(i)=\left\{ \begin{array}{cc} 1, & \text{ if }t_{x}\geq \frac{1}{2}\left(t_{i-1}+t_{i}\right)\\ 0, & \text{ otherwise } \end{array}\right.$$

and

(12) $${\mathrm{surv}}_{f}(x)(i)=0.$$

The dot product in the loss function is used to check the similarities between the predicted and the ground truth vectors.

There are four hyperparameters for training the survival network in this study: number of time intervals, number of layers, number of neurons in each layer, and the learning rate. We optimize the result with the grid search method [48] to obtain the best hyperparameters. We specify feasible hyperparameter ranges, attempt to train the survival network with various hyperparameter combinations, and then evaluate its performance using the AUC on the validation subset.

## 5 Experimental setup

### 5.1 Dataset

We use the KiTS21 dataset [49] for training and evaluating the proposed methodology. KiTS21 comprises diverse information, including tabular data, whole abdominal CT scans, and ground-truth segmentation inundations for the tumors, cysts, and kidneys for 300 unique patients. The patients included in the dataset underwent partial or full nephrectomy for suspected kidney cancer between 2010 and 2020 at M Health Fairview and Cleveland Clinic. The CT images are stored in the NIFTI format, and the tabular data is available in a JSON file format, featuring 63 clinical parameters for each patient [50]. This paper utilizes the whole abdominal CT scans and ground-truth annotations of the tumors and kidneys from the image files. From the tabular data, we only use three variables: 1) ISUP class, 2) event, and 3) time to event. However, we exclude 56 cases with no ISUP value, leaving us with 244 patients to analyze. The “event” field serves as an indicator, with a value of one indicating that an event of interest, death in our study, has occurred for a patient, while a value of zero signifies that the patient has been censored. Out of 244 patients in the dataset, 32 have died, and 212 have censored time. The median observation time is 644 days, and the maximum observation time is 3000 days.

### 5.2 Data splitting

We used three-fold cross-validation to train the classification and survival network. We divided our dataset into three folds depending on the number of dead and censored patients. We desire training and test data to include the same number of dead individuals. Due to the small sample size (244 patients) and class imbalance (ratio of deceased patients to censored patients) in our dataset, we utilized three-fold cross-validation to ensure sufficient deceased patients in each fold for robust model training and testing. With three-fold cross-validation, we could allocate 10 deceased patients to each fold. In contrast, 5-fold or 10-fold cross-validation would have resulted in folds with only 6 or 3 deceased patients, respectively, which was inadequate.

Our goal was to split the data into two main groups - a training plus validation set and a holdout test set for final evaluation. We allocated 33% of the total data to the test set in order to have a suitably sized holdout set. This left 67% for the training and validation data. From the remaining 67%, we allocated 10% to the validation set, leaving 57% for the training set. This division provided a sufficient validation set while maximizing the data available for training the model.

Critically, approximately 12% of the total patients were deceased. To ensure equal representation across all sets, we evenly divided the deceased patients, allocating 4% to the training set, 4% to the validation set, and 4% to the test set. This resulted in the training set containing around 10% deceased patients, the validation set containing around 33%, and the test set containing around 13%. This intentional imbalance ensured enough deceased patients were present in each set to properly develop and evaluate the model's ability to predict survival. The consistent ratios across folds also avoided introducing data leakage or bias. Through this controlled splitting, we aimed to optimize the model training while preserving a suitable test set for unbiased final evaluation of survival prediction accuracy.

In order to guarantee the consistency and reliability of our results, we adopted an identical set of indices for training, validation, and testing in both the classification network and the survival network across all three folds. This indicates that the same data partitions utilized for training, validating, and evaluating the classification network were also utilized for the survival network. This approach maintains the integrity of our evaluation. In this way, we can ensure that the subset used for training the classification network is not used for testing the survival network to prevent bias in our results.

Table 5 delineates the demographic profile of patients from the best-performing fold in the training and validation cohorts, as established by the performance metrics. This fold was selected for its superior predictive accuracy in survival curves as detailed in Section 6.2. It enumerates patient counts, average age with standard deviation, gender distribution, distribution of ISUP grades, and mean survival times with their standard deviations. The demographics of this particular fold are instrumental in illustrating the patient characteristics that contributed to the fold's optimal performance in the study.

**Table 5:** Patient demographics.

Characteristics	Training	Validation
**Number of patients**	162	82

**Age (yr, mean** ± **s.d.)**	60.91 ± 12.64	56.18 ± 13.03

**Sex (n male)**	109 (67%)	52 (63%)

**ISUP grade**		
1	24 (15%)	9 (11%)
2	79 (48%)	40 (49%)
3	43 (27%)	23 (28%)
4	16 (10%)	10 (12%)

**Survival time (days, mean)**	830.74	887.87

**Survival time (days, s.d.)**	741.97	803.60

### 5.3 Evaluation metrics

To evaluate the performance of our survival model, we use three metrics: C-index, the cumulative dynamic area under the curve (AUC), and the integrated Brier score (IBS). The C-index is a rank correlation metric between predicted risk scores and observed time points. It is defined as the ratio of concordant pairs to comparable pairs. If the subject with the shorter observed time encounters an event, then the two samples are comparable. Two pairs are concordant if the estimated risk by a survival model is higher for subjects with lower survival time [51]. C-index is a number between 0 and 1. A value of 0.5 implies random concordance, whereas a value of 1 shows ideal concordance between predicted risk and actual survival [51]. It has been demonstrated that the C-index is overly optimistic given a large number of censored patients in a dataset [52].

We can use cumulative dynamic AUC [53] to understand whether the C-index is optimistic or not. Given a time point *t*, we can estimate how well a predictive model can distinguish subjects who will experience an event by time *t* (sensitivity) from those who will not (specificity). The ROC curve compares the false positive rate (1 - specificity) against the true positive rate (sensitivity). Cumulative dynamic AUC implements an estimator of the cumulative area under the ROC for a given list of time points. AUC is a value between 0 and 1, with 1 being the ideal value [53]. IBS measures the average distance squared between observed survival time and predicted survival probabilities [54]. The model performs better when the IBS is lower, with zero being the best value. To compute the IBS, 100 equally spaced time points are defined between the test dataset's minimum and maximum event timings.

### 5.4 Software and hardware

We developed our software using Python version 3.7.5 from the Anaconda v4.8.2 distribution. The classifier network was trained using PyTorch v1.11.0 on a workstation equipped with a powerful Nvidia GeForce RTX 3090 GPU, an AMD Ryzen 7 5800X 8-Core Processor, and 32 GB of RAM. We developed our survival model by using the pycox v0.2.0.3^3^ library. To measure the evaluation metrics of C-index and IBS, we employed the implementation from pycox v0.2.0.3, and for AUC, we utilized the scikit-survival v0.19.0 library. The input CT images were preprocessed using the MONAI v0.8.1 package. The code created for this project is available at GitHub: Classifaction Part and Survival Part.

## 6 Results and discussion

### 6.1 Performance evaluation by C-index, IBS, and AUC

We evaluated the performance of our survival model using C-index, IBS, and AUC. Using a three-fold cross-validation method resulted in three different numbers for C-index, IBS, and AUC. After that, we took the average of the measures across all three folds to find out how well our survival model worked overall. In the test group, the average C-index, IBS, and AUC were all 0.72, 0.15, and 0.71, respectively. These results demonstrate the model's ability to distinguish between the survival probabilities of different patients.

Table 6 provides an extensive comparison of the C-index for our proposed survival model and numerous related studies. These studies have employed at least one medical imaging modality as input for their organ-specific cancer survival models while also utilizing a specific method for medical image feature extraction. In some of these studies, multiple data modalities were used along with different loss functions. Despite using a non-proportional method, our survival model could surpass all studies based on the CPH method. According to Table 6, the two studies in the second and third rows utilized CT images as inputs. Yet, our survival model outperformed these studies and scored a higher C-index. In contrast, the fourth study used histopathological images, which is an invasive technique. Although histopathological images might contain more survival information, they achieved a lower C-index than our survival model. The fifth, sixth, and seventh studies incorporated CT images and clinical data for their survival analysis. Our survival model outperformed the fifth study, yet it scored lower than the sixth and seventh studies. We attribute these differences in performance to the incorporation of clinical variables, which can provide a richer understanding of the patient's condition compared to CT images alone. The eighth and ninth studies employed genomics data and histopathological images, yet our survival model still yielded a higher C-index score than the former. However, our survival model yielded lower results than the ninth, tenth, and eleventh studies. This is because these studies utilized multiple data modalities as inputs to their survival models, improving performance by enriching the model's comprehension of the patient's condition. Despite utilizing only one data modality as input to our survival model, we were still able to achieve a high C-index.

**Table 6:**
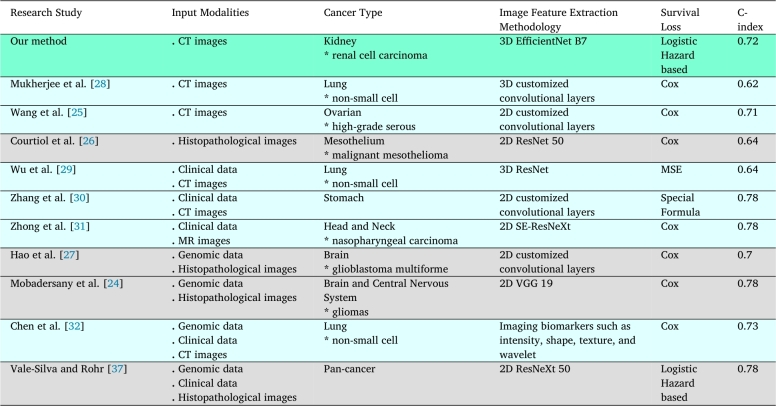
Comparison of this study with previous related studies based on input modalities, cancer type, image feature extraction methodology, survival loss, and C-index.

### 6.2 Performance evaluation by predicted survival curves

**Figure 2:**
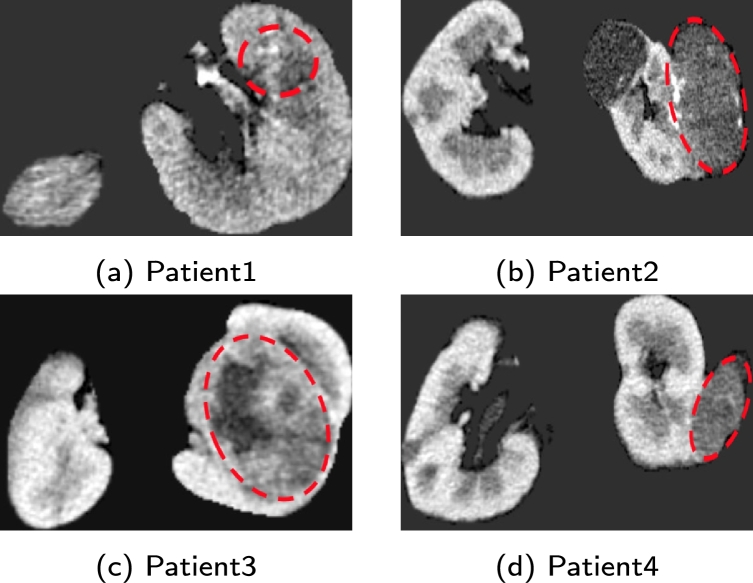
CT images with tumors indicated of patients with event 1.

**Figure 3:**
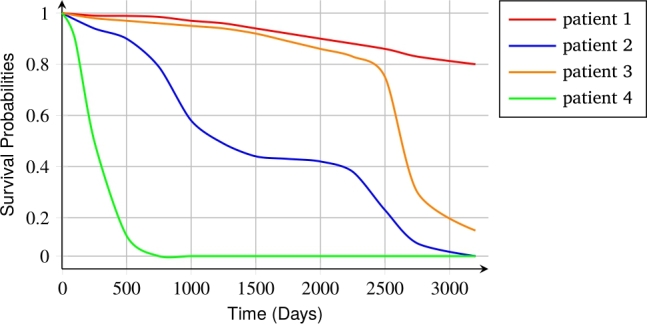
Survival Probabilities for four patients with event 1.

To demonstrate the robustness of our survival model's output, we also plotted survival curves for eight patients in the test subset with varying clinical states and having both censored and uncensored events. Table 7 shows the ISUP grade, event, and time of the event of these patients. Fig. 2 presents the CT images highlighting the tumor locations for four patients who died. The individual parts of Fig. 2, specifically parts (a), (b), (c), and (d), correspond to patients with ISUP grades 1, 2, 3, and 4, respectively. As shown in Fig. 3, our survival model predicts four survival curves for patients 1, 2, 3, and 4, whose events are 1. Patient 4, with an ISUP grade of 4, has the shortest predicted survival time depicted in the green curve, which is the lowest of all the curves. The model indicated that this patient's survival probability would be less than 0.5 after 250 days.

**Table 7:** Information of 8 patients whose survival curves were drawn.

Patient number	ISUP grade	Event	Time (Days)
Patient1	1	1	478
Patient2	2	1	645
Patient3	3	1	1600
Patient4	4	1	48
Patient5	1	0	2044
Patient6	2	0	22
Patient7	3	0	183
Patient8	4	0	303

Despite having an ISUP grade of 2, Patient 2's predicted survival time is shorter than that of Patient 3, with an ISUP grade of 3. Our survival model's prediction for Patient 2 having a lower survival than Patient 3 was accurate. These results show that our survival model was successful in generating survival curves for these four patients. Still, the projected slope might be less steep than the actual slope since predicted survival times could be longer than the actual survival duration. Regarding patient 1, who has ISUP grade 1 and died around 500 days, the model was unable to accurately estimate the survival curve for this patient, instead predicting a high survival chance. The model projected high survival rates for some patients with ISUP grade 1.

**Figure 4:**
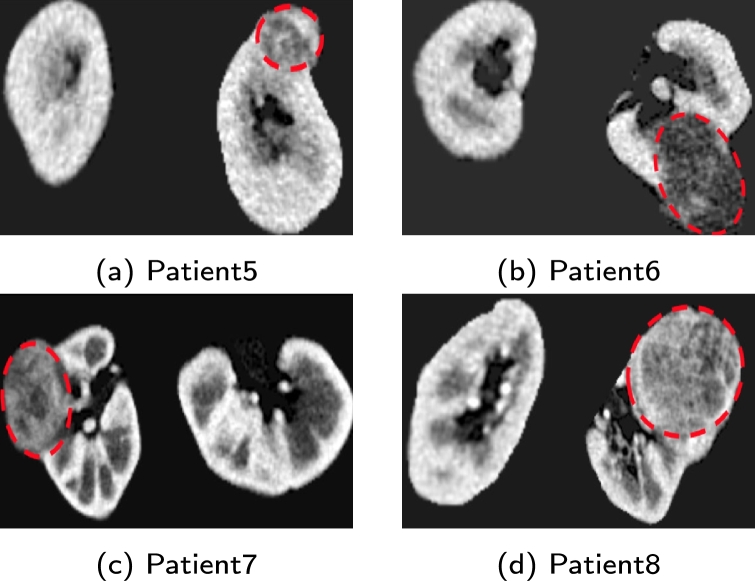
CT images with tumors indicated of patients with event 0.

Fig. 4 shows the CT images with tumors' locations indicated for the four patients who had censored events. The individual parts of Fig. 4, specifically parts (a), (b), (c), and (d), correspond to patients with ISUP grades 1, 2, 3, and 4, respectively.

**Figure 5:**
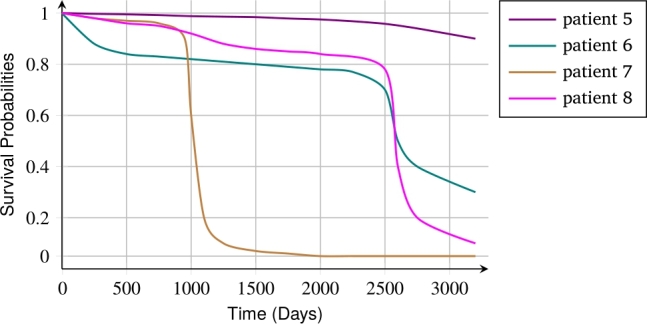
Survival Probabilities for 4 patients whose events were 0.

Our survival model predicts four survival curves for patients 5, 6, 7, and 8, whose event is 0, as shown in Fig. 5. Patient 5, with ISUP grade 1, had the longest censoring time compared to patients 6, 7, and 8.

The predicted survival curve for Patient 5 is higher than the others, and the model predicted a high likelihood of survival for this patient. According to Table 7, Patient 6, with an ISUP grade of 2, had the lowest censoring time. Patient 6's predicted survival curve is lower than patient 7's until around 1000 days and patient 8's until around 2600 days. Patient 7 had a lower predicted survival curve than patients 6 and 8 after 1000 days.

Despite a higher ISUP grade than patients 6 and 7, patient 8, with a longer censoring time, did not have the lowest survival curve, as our survival model correctly predicted. After roughly 1000 days for patient 7, 2600 days for patient 8, and 2700 days for patient 6, our survival model predicted that their survival probability would be less than 0.5.

### 6.3 Study limitations

This study has several limitations: 1) we have two separate networks. The survival network is dependent on the features extracted by the classifier network. A unified network for prognostic feature extraction and survival estimation yielded poor results, likely due to DL models' difficulty in predicting survival time directly from images. However, the accuracy of our survival analysis improved by using features obtained from RCC ISUP grade classification in CT images. Consequently, we independently trained the classifier network for feature extraction and the survival network for survival estimation. 2) In addition to the whole abdomen image, segmentation annotations of the organ of interest and its tumors are required for accurate feature extraction. 3) To extend this study's findings to other organs, it is crucial to identify a clinical variable analogous to ISUP grade that facilitates tumor classification related to survival estimation. 4) The exploration for an alternative publicly accessible dataset, comprising both organ and tumor segmentation as well as pertinent clinical variables (such as ISUP grading or TNM stage) for classification purposes, yielded no results. Consequently, the validation of our methodology with an additional dataset was not feasible. 5) The other is the potential for biases within our training dataset. As indicated in Table 5, a majority of the patients in both the training and testing datasets were categorized under ISUP grade 2. Therefore, our model's performance may vary in external populations with different ISUP grade distributions, potentially affecting the survival analysis outcomes. Furthermore, our study was characterized by a high proportion of censored data. Variations in the amount of censored data in other datasets could lead to discrepancies in survival performance, given that the model's predictions are partly shaped by the censoring patterns observed in the training set.

### 6.4 Future works

There are several directions in which our work can be extended. Firstly, we aim to expand the method by incorporating new input features, image modalities, and data modalities, such as histopathological images, clinical and genomics data. Secondly, we will investigate the feasibility of either merging RCC ISUP grade classification and survival networks for simultaneous training or developing a novel end-to-end survival model without the need for separate tumor grading. Thirdly, we are dedicated to enhancing interpretability for both the classifier and survival networks to gain insights into how the tumors are classified and how the survival probability curves are predicted from the extracted feature vector. Fourthly, we intend to focus on validating the proposed approach in multicenter studies, expanding the dataset to include patients from various demographic backgrounds. Finally, we aim to investigate novel techniques for feature extraction that do not require organ and tumor annotations.

## 7 Conclusion

Our study demonstrates the potential of a deep learning approach to predict survival probabilities of patients with renal cell carcinoma (RCC) based solely on preoperative CT imaging. The application of RCC ISUP grading as a predictor of kidney cancer mortality led to its adaptation in RCC survival analysis, with our findings supporting its potential for facilitating such analysis. By employing various procedures and novel methodologies, such as concatenation and Efficient-Net B7 architecture, we successfully extracted the intrinsic survival characteristics of RCC from ISUP grade classification. We developed a survival network to predict personalized survival curves with a high C-index. Our proposed approach utilizing discrete LogisticHazard-based loss produced more realistic survival curves than Cox regression studies. The results demonstrate that deep learning can yield CT-based prognostic biomarkers for RCC that are as informative as clinical variables. Our study is the first to estimate non-proportional and non-linear survival estimation from CT images solely, improving prognostic biomarker discovery and offering directions for future research in improving prognostic accuracy and patient outcomes in medical practice.

## CRediT authorship contribution statement

**Maryamalsadat Mahootiha:** Writing – review & editing, Writing – original draft, Visualization, Validation, Software, Resources, Methodology, Investigation, Formal analysis, Data curation, Conceptualization. **Hemin Ali Qadir:** Writing – review & editing, Writing – original draft, Visualization, Supervision, Software, Resources, Methodology. **Davit Aghayan:** Writing – review & editing, Validation, Supervision. **Åsmund Avdem Fretland:** Writing – review & editing, Supervision. **Bjørn von Gohren Edwin:** Writing – review & editing, Supervision. **Ilangko Balasingham:** Writing – review & editing, Supervision, Resources, Project administration, Funding acquisition, Conceptualization.

## Declaration of Competing Interest

The authors declare that they have no known competing financial interests or personal relationships that could have appeared to influence the work reported in this paper.

## Data Availability

Given that this dataset is publicly available, there was no need for us to acquire additional patient consent to utilize it in our research paper. It's presumed that the necessary consent was already procured by the individuals who created the dataset. For making this dataset, the University of Minnesota Institutional Review Board granted approval for a retrospective study. The dataset can be used in alignment with the stipulations of the Creative Commons Attribution-NonCommercial-ShareAlike 4.0 International (CC BY-NC-SA 4.0) license [49]. The data utilized for this research is publicly accessible via GitHub at the following link: Kits21 GitHub.

## Footnotes

1 https://docs.monai.io/en/stable/transforms.html.

2 right-anterior-superior.

3 https://github.com/havakv/pycox.
